# Using HPC for teaching and learning bioinformatics software: Benefits and challenges

**DOI:** 10.1186/1471-2105-14-S17-A18

**Published:** 2013-10-22

**Authors:** Tyler Parke, Mark Farman, Elizabeth Farnsworth, Derek Fox, Jerzy W Jaromczyk, Jolanta Jaromczyk, Neil Moore, Christopher L Schardl, Ruriko Yoshida, Pat Calie

**Affiliations:** 1 Department of Computer Sciences, University of Kentucky, Lexington, KY 40506, USA; 2 Department of Plant Pathology, University of Kentucky, Lexington, KY 40546, USA; 3 Advanced Genetic Technologies Center, University of Kentucky, Lexington, KY 40546, USA; 4 Department of Statistics, University of Kentucky, Lexington, KY 40536, USA; 5 Department of Biological Sciences, Eastern Kentucky University, Richmond, KY 40475, USA

## Background

We present our work on using the XSEDE high-performance computing (HPC) network to support and facilitate hands-on bioinformatics tasks for participants of our Essentials of Next Generation Sequencing (NGS) workshop, as well as for students and other learners. In the summer of 2012, the University of Kentucky hosted the NGS workshop, attended by faculty and students from across the Commonwealth who were introduced to the laboratory and bioinformatic components of next-generation sequencing and sequence analysis. Participants used next-generation technology to sequence real genetic material, then used a variety of bioinformatics software tools to assemble those sequences, compare and align them to other sequences, predict genes, and visualize the genome. Due to the success of the 2012 workshop, the second workshop, planned for this summer, is expected to be larger in scale and to include even more participants. It will furthermore include several additional bioinformatics tools and tasks. Since participants will be simultaneously running intensive bioinformatics computing tasks, the resources required will exceed the capacity of the single twelve-core server used to support the workshop last year. One particular resource that appears promising to meet our intensive computational needs is the XSEDE grid computing network, a follow-on to the TeraGrid project designed specifically for "e-Science" and scientific computing. Many of the systems in the XSEDE network already support some of the software used within our workshop; however, many of the programs we will demonstrate have not previously been installed on tested on the XSEDE network. We will describe our experiences porting these applications to, and deploying them on, XSEDE. We will also discuss the challenges that the HPC approach presents for teaching and learning, particularly the complexities of navigating between time-sharing systems and remote job scheduling.

